# Association of Peer Comparison Emails With Electronic Health Record Documentation of Cancer Stage by Oncologists

**DOI:** 10.1001/jamanetworkopen.2020.15935

**Published:** 2020-10-06

**Authors:** Anna D. Sinaiko, Michael L. Barnett, Marema Gaye, Margaret Soriano, Therese Mulvey, Ephraim Hochberg

**Affiliations:** 1 Department of Health Policy and Management, Harvard T. H. Chan School of Public Health, Boston, Massachusetts; 2 Division of General Internal Medicine and Primary Care, Department of Medicine, Brigham and Women’s Hospital, Boston, Massachusetts; 3 Patient Care Services, Massachusetts General Hospital, Boston; 4 Division of Hematology and Oncology, Department of Medicine, General Hospital Cancer Center and Harvard Medical School, Boston, Massachusetts

## Abstract

**Importance:**

Systematically capturing cancer stage is essential for any serious effort by health systems to monitor outcomes and quality of care in oncology. However, oncologists do not routinely record cancer stage in machine-readable structured fields in electronic health records (EHRs).

**Objective:**

To evaluate whether a peer comparison email intervention that communicates an oncologist’s performance on documenting cancer stage relative to that of peer physicians was associated with increased likelihood that stage was documented in the EHR.

**Design, Setting, and Participants:**

This 12-month, randomized quality improvement pilot study aimed to increase oncologist staging documentation in the EHR. The pilot study was performed at Massachusetts General Hospital Cancer Center from October 1, 2018, to September 30, 2019. Participants included 56 oncologists across 3 practice sites who treated patients in the ambulatory setting and focused on diseases that use standardized staging systems. Data were analyzed from July 2, 2019, to March 5, 2020.

**Interventions:**

Peer comparison intervention with as many as 3 emails to oncologists during 6 months that displayed the oncologist’s staging documentation rate relative to all oncologists in the study sample.

**Main Outcomes and Measures:**

The primary outcome was patient-level documentation of cancer stage, defined as the likelihood that a patient’s stage of disease was documented in the EHR after the patient’s first (eg, index) ambulatory visit during the pilot period.

**Results:**

Among the 56 oncologists participating (32 men [57%]), receipt of emails with peer comparison data was associated with increased likelihood of documentation of cancer stage using the structured field in the EHR (23.2% vs 13.0% of patient index visits). In adjusted analyses, this difference represented an increase of 9.0 (95% CI, 4.4-13.5) percentage points (*P* = .002) in the probability that a patient’s cancer stage was documented, a relative increase of 69% compared with oncologists who did not receive peer comparison emails. The association increased with each email that was sent, ranging from a nonsignificant 4.0 (95% CI, −0.8 to 8.8) percentage points (P = .09) after the first email to a statistically significant 11.2 (95% CI, 4.9-17.4) percentage points (P = .003) after the third email . The association was concentrated among an oncologist’s new patients (increase of 11.8 [95% CI, 6.2-17.4] percentage points; *P* = .001) compared with established patients (increase of 1.6 [95% CI, –2.9 to 6.1] percentage points; *P* = .44) and persisted for 7 months after the email communications stopped.

**Conclusions and Relevance:**

In a quality improvement pilot trial, peer comparison emails were associated with a substantial increase in oncologist use of the structured field in the EHR to document stage of disease.

## Key Points

**Question:**

Is an email intervention to communicate an oncologist’s performance in electronic health record (EHR) documentation of cancer stage in comparison with peer physicians associated with increased likelihood of documentation?

**Findings:**

In a randomized quality improvement pilot study of 56 oncologists, emails with peer comparison data on the proportion of an oncologist’s patients with stage documented in the electronic health record (EHR) were associated with increased documentation of patients’ stage in the EHR by 9.0 percentage points, a relative increase of 69%. The association was observed among oncologists’ new, but not established, patients.

**Meaning:**

These findings suggest that quality improvement interventions using peer comparisons could change use of the EHR among oncologists.

## Teaser

This quality improvement pilot study evaluates whether an email intervention that communicates an oncologist’s performance in documenting cancer stage relative to that of peer physicians is associated with increased likelihood that stage is documented in the electronic health record (EHR).

## Introduction

For patients with solid tumors of a given histologic type and primary site, cancer stage is the single most important determinant of treatment approach and survival, directly informing the choice of an active treatment plan. Cancer stage is also used to identify patients eligible for clinical trials or those in need of multimodality care or conversations about serious illness, all practices that are associated with higher-quality and higher-value care.^1^ Systematically capturing cancer stage is essential for any serious effort by health systems to monitor outcomes and quality of care in oncology.

Unfortunately, oncologists do not routinely record cancer stage in machine-readable structured fields in electronic health records (EHRs).^2^ Documenting staging in a structured field involves more of an oncologist’s time to complete additional clicks in the patient’s EHR without any additional compensation. It also requires a change in clinical documentation process from that of prior practice. Both of these requirements can be barriers to clinician adoption of EHR use.^3,4^ However, there are no clear alternatives to structured fields for systematically collecting staging data. Free text in a problem list or a clinical note is very difficult to extract on a system level,^2^ whereas natural language processing to automate free-text review requires unique algorithms for each disease group that can also be error prone.^2,5^

The absence of routine cancer stage documentation is a barrier to expand quality improvement efforts in oncology, making a case for programs or additional incentives to encourage clinicians to complete staging documentation. Possible interventions include directed education, sharing information with oncologists about their documentation rates (peer comparison), providing financial incentives to reward staging documentation, or some combination of the three. Evidence on whether these interventions are effective at achieving increased staging documentation is mixed. Education alone is often not an effective means to achieve widespread adoption of quality of care best practices or to encourage changes in aggregate physician behavior.^6,7^ Financial incentives, which are often used within health systems to reward physicians on a variety of metrics, have a mixed track record.^8,9^ On the other hand, peer comparison interventions, where physicians are presented with their performance relative to that of a peer group, are low cost and have been shown to result in greater physician adherence to guideline-recommended care in nononcology clinical contexts.^10,11^ Designing and framing peer comparisons in ways that leverage findings from behavioral economics to motivate change has potential to achieve an even greater impact.^12^

At our institution, a cancer center at a large academic hospital, the need to evaluate outcomes of new clinical initiatives made documentation of cancer stage essential. We applied insights from behavioral science to design and implement a quality improvement intervention using peer comparison to increase rates of cancer stage documentation. We evaluated the association of this initiative with staging documentation for 12 months, including a 6-month pilot phase and a subsequent 6-month follow-up period.

## Methods

### Design

For this quality improvement study, we randomly assigned oncologists to either receive 3 separate emails with individualized data on their cancer stage documentation activity compared with their peers or to receive no email communication during a 12-month pilot intervention period. Because the study was part of quality improvement activities to improve staging documentation, the institutional review board at the Harvard T. H. Chan School of Public Health, Boston, Massachusetts, determined that the study did not meet the definition of human research and was not subject to review; thus, no consent was sought from study participants. This study conformed with the Standards for Quality Improvement Reporting Excellence (SQUIRE) reporting guidelines for quality improvement studies. In addition, we retrospectively registered this study as a clinical trial at clinicaltrials.gov because the study could be interpreted as a clinical trial.^13^

### Setting and Study Participants

The study setting was the Massachusetts General Hospital Cancer Center (MGHCC), consisting of 3 campuses located in Boston, Danvers, and Waltham. The study period was January 1, 2018, to September 30, 2019, divided into a 6-month lookback period (January 1 to June 30, 2018), a prestudy period (July 1 to September 27, 2018), and an intervention period (October 1, 2018, to September 30, 2019). Massachusetts General Hospital Cancer Center implemented and has used the Epic Electronic Medical Record across all of these sites since April 2016.

**Figure 1.**
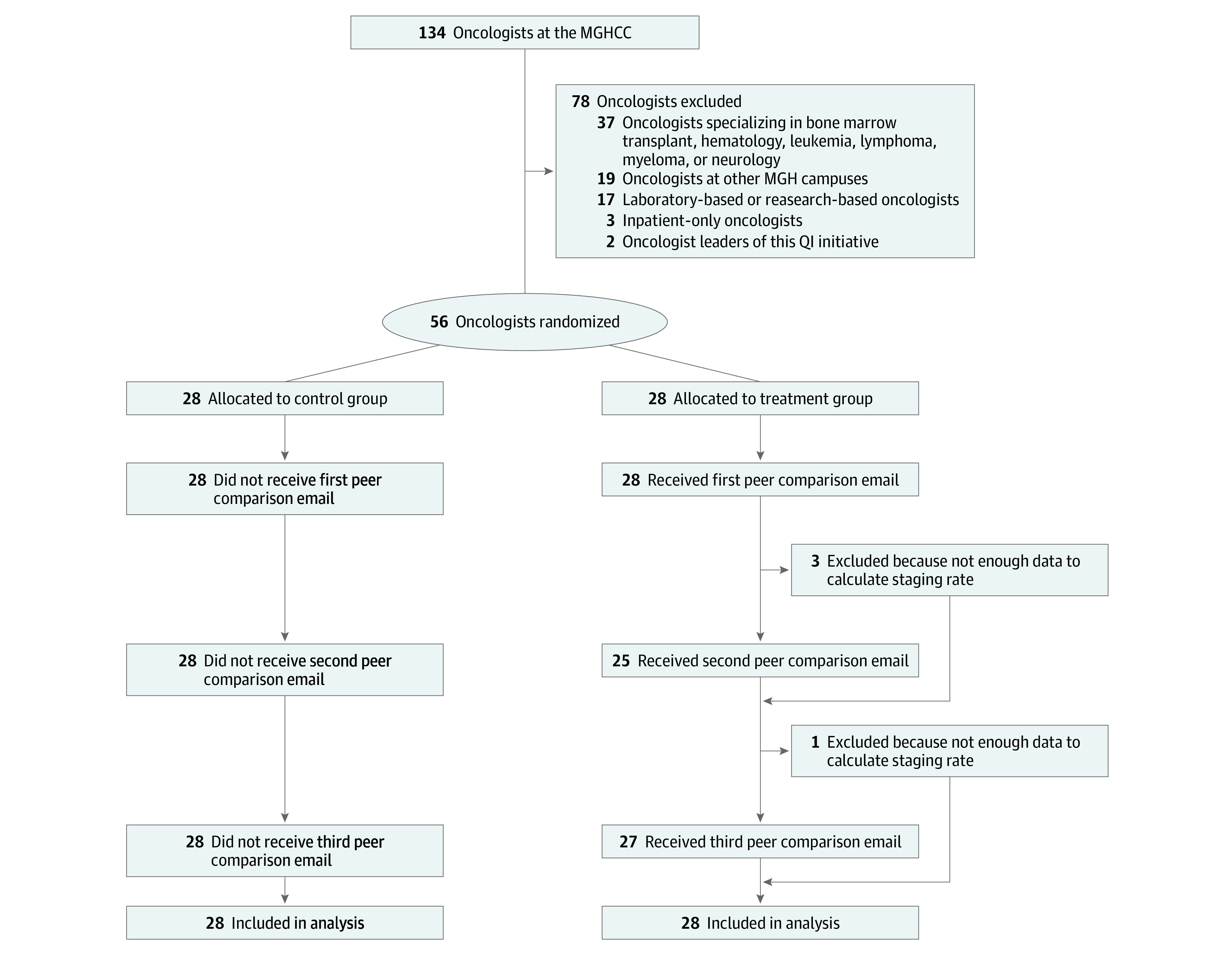
Cohort Construction MGHCC indicates Massachusetts General Hospital Cancer Center; QI, quality improvement.

The physicians included in the pilot were the full population of eligible attending oncologists. All oncologists had been at 1 of these 3 sites for a minimum of 9 months, including the lookback and the prestudy periods, which we required to determine each oncologist’s new vs established patients. We excluded oncologists treating patients with cancer for which American Joint Committee on Cancer staging modules were less clinically relevant (hematology, bone marrow transplant, leukemia, lymphoma, myeloma, and neuro-oncology disease groups), those delivering care at other locations, those with low clinical volume (<5 patients during 3 months) or no outpatient visits (ie, inpatient only or primary research faculty), and those leading this quality improvement initiative. The final sample included 56 physicians; 49 were oncologists with a specific disease focus (ie, breast, gastrointestinal tract, genitourinary, gynecology, head and neck, melanoma, sarcoma, or thoracic cancers), and 7 were general medical oncologists treating a variety of solid tumors. All patients with at least 1 outpatient visit during the study period with 1 of the study oncologists were included (Figure 1).

### Interventions

In September 2018, the MGHCC rolled out information about a quality improvement initiative to increase documentation of staging, and MGHCC leadership communicated the importance of staging documentation in the EHR to all oncologists during disease group or practice site–wide meetings. Starting in October 2018, emails were sent during a 6-month randomized quality improvement pilot, with a random half of eligible oncologists assigned to receive emails containing individualized peer comparison data.

**Figure 2.**
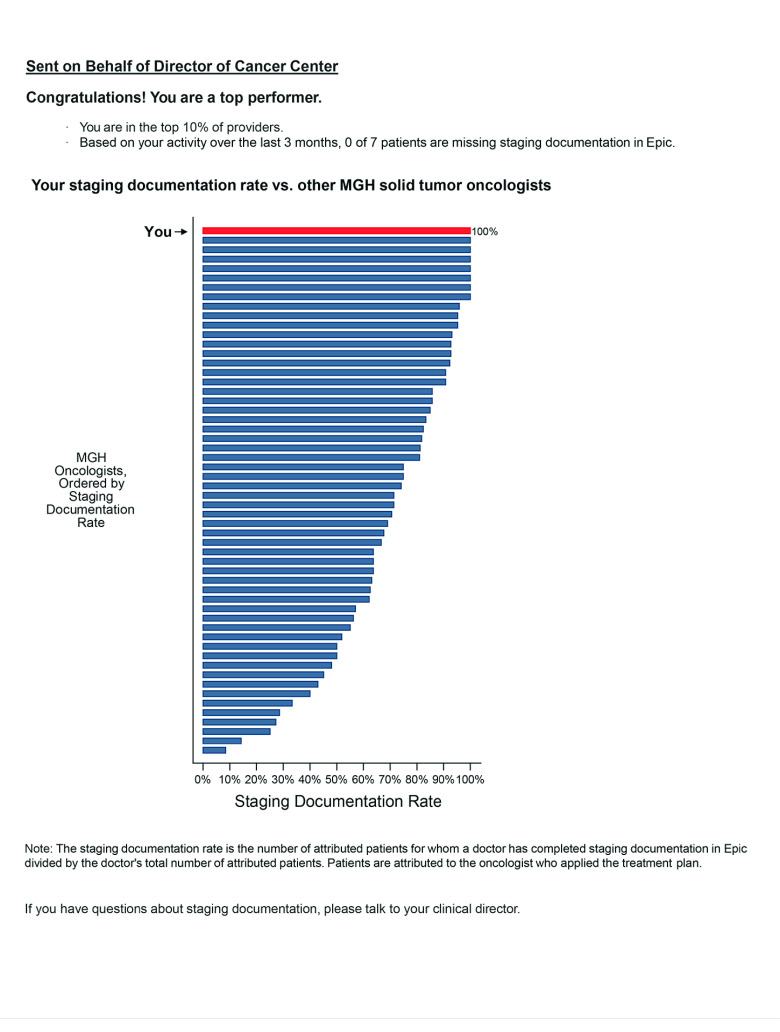
Sample Peer Comparison Intervention Email for Top Performers MGH indicates Massachusetts General Hospital.

Specifically, the emails contained a chart showing the oncologist information about his or her own staging documentation rate relative to all other included oncologists. Staging documentation rate was calculated as the percentage of an oncologist’s patients for whom disease stage was documented in the structured field in the EHR. The email text followed methods used previously in a similar intervention to reduce inappropriate antibiotic prescribing.^10^ Oncologists with the highest staging documentation rate (the top performing decile or all oncologists with a rate of 100%, whichever was larger) received an email in which both the subject line and email text said they were a top performer. All other pilot oncologists received emails saying they were not a top performer. The emails included a chart showing the distribution of staging documentation rates across MGHCC oncologists in the sample, deidentified for all oncologists except for the oncologist recipient (see Figure 2 for sample email).

Emails were sent from MGHCC leadership in 3 waves: first on September 28, 2018 (using data from visits from May 1 to July 31, 2018), then November 8, 2018 (using data from visits from October 1 to 31, 2018), and finally on January 11, 2019 (using data from visits from November 1 to December 31, 2018). Oncologists received up to 3 emails: a minimum of 3 eligible patient visits during each reporting period was required for an oncologist to receive an email (Figure 1).

### Outcomes

The primary outcome for this pilot was staging documentation using the structured field in the EHR, which was measured in 2 ways using data elements extracted from the EHR. Patient-level staging documentation was measured as an indicator of whether the structured field for stage in the patient’s EHR was populated within 28 days of the date of the first outpatient or index encounter from July 1, 2018, through September 30, 2019. This was the patient’s first encounter during the study period (eg, the prestudy and the intervention periods).

We also measured oncologist-level rates of staging documentation, calculated as the percentage of an oncologist’s patients for whom disease stage was documented in the structured field in the EHR. Staging documentation rate was calculated monthly using all patients with an outpatient encounter in 1 month as the denominator. Patients are attributed to the oncologist who applied the treatment plan.

### Randomization Approach

The oncologists included in the peer comparison pilot were selected randomly using an optimal nonbipartite matching function, conducted using the R statistical programming package nbpMatching, version 3.5.2 (R Institute).^14^ This function matched oncologists into pairs that maximized the similarity of each pairs’ patient volume and percentage of patients with documented staging in an earlier 3-month period (May 1 to July 31, 2018). Within each pair, one oncologist was then randomly assigned to participate in the pilot intervention and one was not. This process was repeated 100 times, and the randomizations were assessed for balance on disease group between pilot participants and nonparticipants. The randomization with the best balance was selected to control for the differential use of staging between 2 sets of patients with dramatically different disease characteristics (eg, breast cancer and sarcoma) and to more accurately compare the impact of the intervention within the unique culture of each disease group.

### Statistical Analysis

Data were analyzed from July 2, 2019, to March 5, 2020. We compared mean (eg, unadjusted) rates of staging documentation between the pilot group and the comparison group and used multivariable linear regression models to evaluate the pilot. We estimated patient-level models; the unadjusted model included an indicator for whether the oncologist received peer comparison emails, an indicator for whether the patient’s index encounter occurred during the intervention, and the interaction between the two, which was the key estimate of interest. A second model adjusted for physician characteristics, including disease focus, annual productivity (measured using quartile of relative value units generated per physician in the 2018 fiscal year), tenure in practice (measured using quartile of years since medical school graduation), and physician sex. To account for intraphysician and intradisease group correlation, all models used robust standard errors clustered at the disease-group level.

To test for remaining unobservable differences between pilot participants and nonparticipants, we performed models that included physician fixed effects to control for all time-invariant observable and unobservable within-physician differences. In addition, because the dependent variable in all models was binary (whether a patient’s disease stage was documented on his or her first encounter with an MGHCC physician), we conducted sensitivity analyses using logistic regression. Results from sensitivity analyses are similar (eTable in the Supplement).

To examine variation in oncologist responses, we conducted 2 additional analyses. First, we examined whether the association between the intervention and oncologists’ responses changed over time by including separate indicators that measured when the patient encounter occurred relative to the timing of each email and interacting each of these indicators with oncologist receipt of peer comparison emails. Second, we analyzed the probability of stage documentation separately for each oncologist’s new patients vs for established patients. New patients were defined as patients who had no encounter with the MGHCC during the 6-month lookback period (ie, clean period) from January 1 to June 30, 2018, which was just before the prestudy period. Established patients were all remaining patients who by definition had 1 or more encounters with a study oncologist during this lookback period. We measured whether the structured field for stage in the patient’s EHR was populated within 28 days of each patient’s index encounter. For new patients, this completed field was their first encounter, whereas for established patients, this was the first encounter since the start of the prestudy period. To adjust for multiple comparisons, we applied the Bonferroni correction, and 2-sided *P* < .008 was considered statistically significant.

## Results

### Sample Characteristics

Fifty-six oncologists (32 men [57%] and 24 women [43%]) were eligible to receive peer comparison emails; these physicians had 23 226 patient encounters during the prestudy and intervention periods. The oncologists who received vs did not receive peer comparison emails were balanced on distribution across disease group, annual productivity, sex, mean number of years since medical school graduation, and mean number of attributed patients and visits (Table 1). Before the intervention, there was no difference in staging documentation rates of oncologists in the pilot vs the comparison groups.

**Table 1.** Physician Characteristics in Prestudy Period^a^

Characteristic	Control group (n = 28)	Treatment group (n = 28)	*P* value^b^
Sex			
Female	14 (50)	10 (36)	.21
Male	14 (50)	18 (64)	
Disease group			
Breast	6 (21)	6 (21)	>.99
Danvers, Massachusetts, only	2 (7)	4 (14)	
Gastrointestinal	5 (18)	6 (21)	
Genitourinary	2 (7)	2 (7)	
Gynecology	2 (7)	1 (4)	
Head and neck	2 (7)	1 (4)	
Melanoma	2 (7)	2 (7)	
Sarcoma	1 (4)	1 (4)	
Thoracic	5 (18)	5 (18)	
Waltham, Massachusetts, only	1 (4)	0	
Relative value unit quartile			
1 (lowest)	7 (25)	6 (21)	.88
2	6 (21)	8 (29)	
3	6 (21)	7 (25)	
4 (highest)	8 (29)	5 (18)	
Missing	1 (4)	2 (7)	
Time since graduation from medical school, mean (SD), y	19 (10)	21 (12)	.63
Total No. of unique patients in prestudy period, mean (SD)	174 (88)	169 (102)	.67
Total No. of office visits in prestudy period, mean (SD)	275 (124)	258 (117)	.45

^a^ Indicates 3 months before peer comparison intervention (July 1 to September 27, 2018). Unless otherwise indicated, data are expressed as number (percentage) of oncologists.

^b^ Differences were calculated using Fisher exact test for disease group and relative value unit quartile; Wilcoxon rank sum test, for years since graduation from medical school, total number of unique patients in the prestudy period, and total number of office visits in the prestudy period.

### Staging Documentation

During the intervention, oncologists who received peer comparison emails documented cancer stage in the structured field within 28 days of a patient’s index encounter more frequently than oncologists who did not receive the emails (23.2% vs 13.0% of patient index visits) (Table 2). In unadjusted models, receipt of the peer comparison emails by a patient’s oncologist was associated with an increase of 9.5 (95% CI, 4.2-14.8) percentage points (*P* = .003) in the probability that the patient’s cancer stage was documented using the structured field. Controlling for measured physician differences in disease focus, relative value unit productivity, sex, and tenure decreased this estimate to 8.2 (95% CI, 2.3-14.0) percentage points (*P* = .01). In models that included physician fixed effects, which control for all (ie, observed and unobserved) differences between oncologists, the associated increase was 9.0 (95% CI, 4.4-13.5) percentage points (*P* = .002). Compared with the staging documentation rate of 13.0% among oncologists who did not receive the peer comparison emails, this difference represented a relative increase of 69%. The increase in probability of a patient’s stage being documented was greater with each email wave, ranging from a nonsignificant 4.0 (95% CI, −0.8 to 8.8) percentage points (*P* = .09) after the first email to a statistically significant 11.2 (95% CI, 4.9-17.4) percentage points (*P* = .003) after the third email (Table 2).

**Table 2.** Association of Intervention With Documentation of Staging a Patient’s Cancer Within 28 Days of Index Visit^a^

Patient group	No. of patients in estimates	Documented cancer stage after email, mean %		Difference (95% CI), percentage points					
		Control group	Intervention group	Unadjusted	*P* value	Adjusted^b^	*P* value	Adjusted^c^	*P* value
All	23 226	13.0	23.2	9.5 (4.2 to 14.8)	.003	8.2 (2.3 to 14.0)	.01	9.0 (4.4 to 13.5)	.002
Seen between first and second email	2846	11.7	17.3	4.9 (0.5 to 9.3)	.03	4.8 (–0.6 to 10.3)	.08	4.0 (–0.8 to 8.8)	.09
Seen between second and third email	3300	12.1	21.1	8.4 (0.6 to 16.1)	.04	8.3 (0.1 to 16.5)	.05	8.5 (1.7 to 15.3)	.02
Seen after third email	7481	14.0	26.5	11.8 (5.5 to 18.2)	.002	9.3 (2.5 to 16.2)	.01	11.2 (4.9 to 17.4)	.003
New	11 907	17.6	33.8	11.6 (8.0 to 15.2)	<.001	10.7 (6.8 to 14.6)	<.001	11.8 (6.2 to 17.4)	.001
Established	11 319	5.5	6.3	2.2 (–2.8 to 7.2)	.34	1.3 (–3.7 to 6.4)	.56	1.6 (–2.9 to 6.1)	.44

^a^ Results are contrasts of adjusted estimates from multivariable linear regression models, in which the dependent variable was a binary variable indicating whether a patient’s disease stage was documented within 28 days of his or her first encounter with a Massachusetts General Hospital Cancer Center physician. Two-sided *P* < .008 was considered statistically significant.

^b^ Adjusted for physician characteristics (disease group, time since medical school graduation, productivity, and sex). Office visits of physicians with missing data on characteristics are excluded from the regression models (n = 1749 [846 new patient visits and 903 with established patient visits]).

^c^ Includes individual physician fixed effects to control for all time-invariant observable and unobservable within-physician differences.

**Figure 3.**
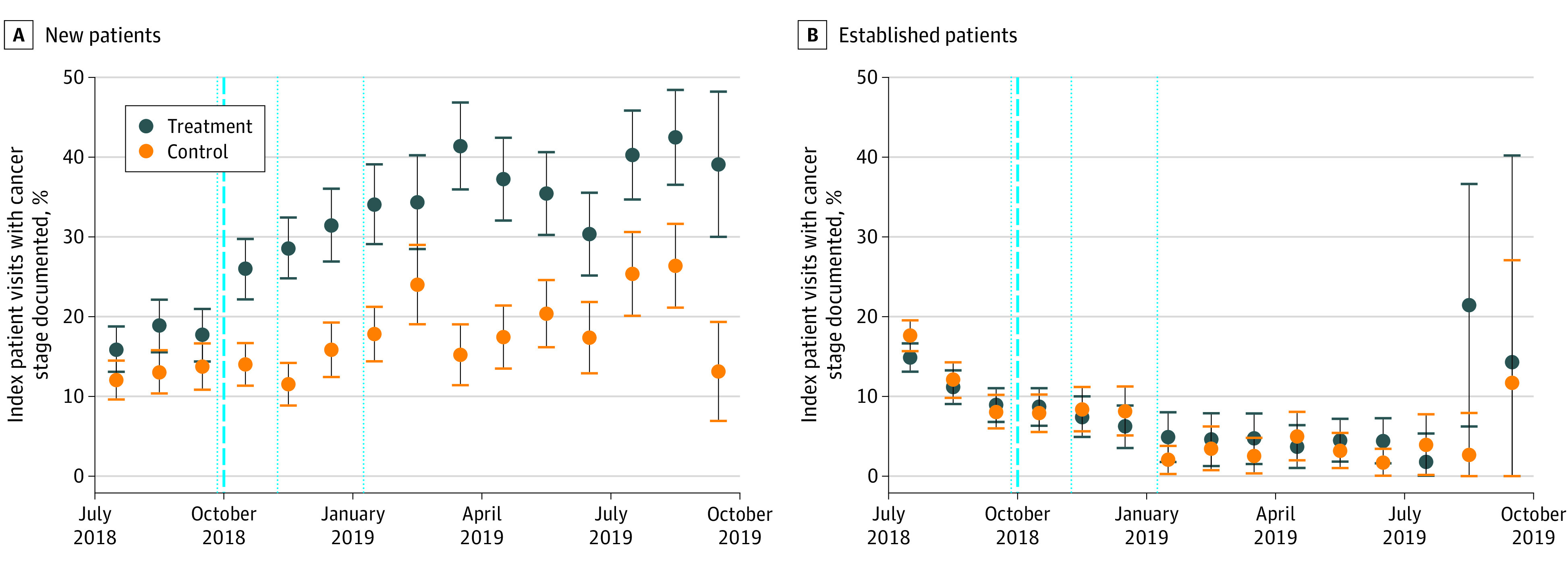
Staging Documentation Rate for New vs Established Patients Data were obtained from July 1, 2018, to September 30, 2019. The data points represent the percentage of index visits with cancer stage documented per calendar month among new patients (A) and established patients (B). New patients had had no encounter with the Massachusetts General Hospital Cancer Center during the 6-month lookback period from January 1 to June 30, 2018. Established patients by definition had 1 or more encounters with a study physician during the lookback period. The error bars indicate Wald 95% CIs. The bold vertical dashed line indicates the first month of the intervention period, October 2018. The dotted vertical lines indicate when physicians in the treatment group received a peer comparison email (September 28, 2018, November 8, 2018, and January 11, 2019). All estimates are unadjusted.

Among new patients, receipt of the peer comparison emails was associated with increased staging documentation by an adjusted difference of 11.8 (95% CI, 6.2-17.4) percentage points (*P* = .001), which represented an increase of 67% over the comparison group (Table 2). This result was observed among new patients having an index visit in any of the 7 months after the last email sent (Figure 3A). Receipt of the peer comparison emails was not associated with increased staging documentation for established patients (increase of 1.6 [95% CI, –2.9 to 6.1] percentage points; *P* = .44) (Figure 3B).

## Discussion

In this pilot quality improvement intervention, delivering peer comparison data to oncologists was associated with an increased likelihood that a patient’s cancer stage was documented in the EHR. Results stratified by new vs established patients showed that the response was focused in oncologists’ new patients. There was no evidence that oncologists receiving emails were more likely to catch up on and complete documentation for established patients. The higher staging documentation rate among new patients persisted for 7 months after the last email, a promising sign that the intervention could have led to durable behavior change. Future evaluation should examine whether results persist going forward.

Consistent with studies of peer comparison interventions to increase preventive screening for colon cancer^15^ and to reduce prescribing of inappropriate medications,^10,16,17^ this study presents evidence that peer comparisons can leverage internal competitiveness of physicians for behavior change. A novel feature of this study is its analysis of the association of peer comparison emails and EHR documentation behavior in contrast to prior literature that has primarily focused on promoting high-quality care delivery (eg, implementation of evidence-based care guidelines). Although EHR documentation can be a burdensome, nonclinical component of medical practice, it plays a fundamental role in modern health care delivery and quality improvement. Our results suggest that peer comparison could be an effective tool to guide clinician behavior in domains beyond patient-directed care.

A few components of this intervention design may have been particularly important. First, we showed oncologists their own individual-level data relative to the top performers as opposed to the mean and median level of performance; peer comparison interventions that use this latter design may not be as effective.^18^ Second, physicians were shown peer comparison information about other oncologists most like themselves—those practicing in the same cancer center and thus treating the same patient population while facing the same system-wide constraints and pressures. Finally, the focus of the intervention was a criterion standard practice (ie, to document staging in the EHR) with a largely motivational barrier impeding completion (ie, the additional time and clicks without any additional compensation). Quality improvement targets with these 2 characteristics may be well suited for peer comparison intervention without leading to negative unintended consequences.^19^

Although we observed a positive association from these peer comparison emails, at the end of the study period, the highest probability of stage of disease documentation was still only 40% among new patients. All other patients were still presumably undergoing staging within free-text fields, but this level of documentation outside of structured fields is far from ideal. Continued efforts are necessary to understand whether this result is observed in other settings and how to increase staging documentation beyond 40% of patients. For example, this quality improvement pilot was only 6 months in duration. Future implementation of similar interventions could distribute peer comparison data for a longer period, especially because this analysis found that each peer comparison email was associated with continued increases in the documented staging rate. Reinforcing interventions implemented at the same time, such as continued messaging at practice meetings, may also be helpful.

### Limitations

This study has several limitations. The intervention involved a small number of oncologists at a single multisite academic oncology practice, and these results may not generalize to other settings. We only tracked outcomes for 7 months after the last email intervention, so we were not able to observe whether the effect associated with the intervention decayed or persisted over time. Given that the study took place in a single academic practice, the comparison group may have been influenced by emails sent to oncologists in the pilot and shared with colleagues. This influence would have theoretically biased our effect toward the null. Finally, as a prospective quality improvement intervention, this study was not preregistered as a randomized clinical trial with a formally prespecified protocol. Therefore, our results should be interpreted as exploratory.

## Conclusions

This study provides evidence that peer comparison feedback is a potential tool to change EHR documentation behavior among physicians. These results can be used to increase health systems’ ability to leverage the EHR to track and measure performance in oncology practices. Of course, peer comparison interventions cannot be applied to all behaviors in need of quality improvement at once. The number of peer comparison interventions that can be deployed at 1 time before leading to adverse consequences, such as burnout or fatigue due to too many peer comparisons (similar to alert fatigue), is unknown, but it could plausibly be 1 or 2. Further research should be conducted to determine how best to deploy this strategy across health systems’ top priorities for quality improvement so that it can be part of the tool kit to improve clinical quality of care.

## Notes

Supplement. **eTable.** Association of Intervention on Staging Documentation of a Patient’s Cancer Within 28 Days of Index Visit With Sensitivity Analysis Using Logistic Regression
