# Supplementary material for: Association of Peer Comparison Emails With Electronic Health Record Documentation of Cancer Stage by Oncologists
Source: JAMA Netw Open. 2020 Oct 6;3(10):e2015935. doi: 10.1001/jamanetworkopen.2020.15935 (PMC7539129; doi:10.1001/jamanetworkopen.2020.15935)
Supplement: Supplement. — eTable. Association of Intervention on Staging Documentation of a Patient’s Cancer Within 28 Days of Index Visit With Sensitivity Analysis Using Logistic Regression [file jamanetwopen-e2015935-s001.pdf]

Supplementary Online Content

Sinaiko AD, Barnett ML, Gaye M, Soriano M, Mulvey T, Hochberg E. Association of peer comparison emails with electronic health record documentation of cancer stage by oncologists. *JAMA Netw Open*. 2020;3(10):e2015935. doi:10.1001/jamanetworkopen.2020.15935

eTable. Association of Intervention on Staging Documentation of a Patient's Cancer Within 28 Days of Index Visit With Sensitivity Analysis Using Logistic Regression

This supplementary material has been provided by the authors to give readers additional information about their work.

©2020 Sinaiko AD et al. *JAMA Network Open*.

eTable. Association of Intervention on Staging Documentation of a Patient's Cancer Within 28 Days of Index Visit With Sensitivity Analysis Using Logistic Regression

				Unadjusted		Model includes controls for physician characteristics		Model includes individual physician fixed effects	
Percent with disease stage documented in EHR	N patients[a]	Post-period Control Mean	Post-period Intervention Mean	Difference (95% CI); percentage pts	*P* value	Adjusted Difference[b] (95%CI); percentage pts	*P* value	Adjusted Difference (95%CI); percentage pts	*P* value
**All patients**	23,226	13.0%	23.2%	9.5 (4.9 - 14.2)	<.001	8.3 (4.0 - 12.6)	p<0.001	7.7 (4.9 - 10.5)	<.001
Patients seen									
Between first and second email	2,846	11.7%	17.3%	5.5 (1.4 - 9.7)	.009	5.8 (0.1 - 11.5)	0.046	3.7 (-0.8 - 8.3)	.109
Between second and third email	3,300	12.1%	21.1%	9.2 (1.7 - 16.6)	.016	9.4 (2.3 - 16.5)	0.010	7.9 (3.0 - 12.9)	.002
After third email	7,481	14.0%	26.5%	12.0 (6.4 - 17.6)	<.001	9.5 (4.7 - 14.3)	p<0.001	9.9 (6.5 - 13.4)	<.001
**New patients**	11,907	17.6%	33.8%	10.3 (5.9 - 14.7)	<.001	9.1 (5.0 - 13.2)	p<0.001	11.0 (6.4 - 15.5)	<.001
**Established patients**	11,319	5.5%	6.3%	1.4 (-0.9 - 3.7)	.231	1.3 (-2.0 - 4.6)	0.439	0.9 (-1.2 - 3.0)	.393

Results are contrasts of adjusted predictions that were estimated from multivariable logistic regression models, where the dependent variable was a binary variable indicating whether a patient's disease stage was documented within 28 days of his/her first encounter with MGH Cancer Center physician. Adjusted predictions generated using STATA.

[a] Indicates the number of patients included in the estimates

[b] Model adjusts for physician characteristics (disease group, time since medical school graduation, productivity, and gender). Office visits of physicians with missing data on characteristics are excluded from the regression models (n=1,749 – 846 new patient visits and 903 with established patient visits).

EHR = Electronic Health Record; 95% CI = 95% Confidence Interval; MGH = Massachusetts General Hospital

©2020 Sinaiko AD et al. *JAMA Network Open*.
